# Gas Chromatography–Mass Spectrometry (GC–MS) Metabolites Analysis in Endometriosis Patients: A Prospective Observational Translational Study

**DOI:** 10.3390/jcm12030922

**Published:** 2023-01-24

**Authors:** Stefano Angioni, Francesca Congiu, Salvatore Giovanni Vitale, Maurizio Nicola D’Alterio, Antonio Noto, Giovanni Monni, Maria Laura Santoru, Vassilios Fanos, Federica Murgia, Luigi Atzori

**Affiliations:** 1 Division of Gynecology and Obstetrics, Department of Surgical Sciences, University of Cagliari, 09124 Cagliari, Italy; 2 ASSL Lanusei, ATS Sardinia, Obstetrics and Gynecology Unit, 08045 Lanusei, Italy; 3 Department of Surgical Sciences, Neonatal Intensive Care Unit, Puericulture Institute, and Neonatal Section, University of Cagliari, 09124 Cagliari, Italy; 4 Department of Prenatal and Preimplantation Genetic Diagnosis and Fetal Therapy, Ospedale Pediatrico Microcitemico A. Cao, 09124 Cagliari, Italy; 5 Department of Biomedical Sciences, Unit of Clinic Metabolomics, University of Cagliari, 09124 Cagliari, Italy

**Keywords:** endometriosis, metabolomics, gas chromatography–mass spectrometry, multivariate analysis, biomarkers

## Abstract

Background: Endometriosis affects women of reproductive age, and its pathogenesis is still unclear. Typically, it overlaps other similar medical and surgical conditions, determining a delay in early diagnosis. Metabolomics allows studying metabolic changes in different physiological or pathological states to discover new potential biomarkers. We used the gas chromatography–mass spectrometer (GC–MS) to explore metabolic alterations in endometriosis to better understand its pathophysiology and find new biomarkers. Methods: Twenty-two serum samples of patients with symptomatic endometriosis and ten without it were collected and subjected to GC–MS analysis. Multivariate and univariate statistical analyses were performed, followed by pathway analysis. Results: Partial least squares discriminant analysis was performed to determine the differences between the two groups (*p* = 0.003). Threonic acid, 3-hydroxybutyric acid, and proline increased significantly in endometriosis patients, while alanine and valine decreased. ROC curves were built to test the diagnostic power of metabolites. The pathway analysis identified the synthesis and degradation of ketone bodies and the biosynthesis of phenylalanine, tyrosine, and tryptophan as the most altered pathways. Conclusions: The metabolomic approach identifies metabolic alterations in women with endometriosis. These findings may improve our understanding of the pathophysiological mechanisms of disease and the discovery of new biomarkers.

## 1. Introduction

Endometriosis is a chronic inflammatory disease characterized by the implantation of the stromal and/or endometrial glandular epithelium outside the uterus [1]. The ectopic endometrium is receptive to the cyclical fluctuation of ovarian steroids in terms of proliferation, differentiation, and bleeding [2,3,4].

It is estimated that endometriosis affects between 10 and 20 percent of women of reproductive age [5], causing dysmenorrhea, dyspareunia, noncyclical pelvic pain, infertility, and consequently, impairment of quality of life, sexual health, and psychological well-being [6,7,8,9,10].

Several hypotheses on its pathogenesis have been postulated: genetic, hormonal, environmental, and immunological factors have been contemplated; however, the exact role of each etiological factor remains unclear [11,12,13].

This complex scenario makes endometriosis a highly heterogeneous disease characterized by infertility and pain with a strong immuno-inflammatory component [14,15]. Indeed, a dysregulated immune response seems to be one of the most critical aetiologic factors related to increased production of pro-inflammatory cytokines, autoantibodies, growth factors, oxidative stress, increased number and activation of macrophages, which are also responsible for adhesion, anatomy changes, scars, and fibrosis [16].

Until now, endometriosis has been underdiagnosed because symptoms are often confused with many other medical and surgical conditions [17]. In addition, the severity of the symptomatology is not related to the extension of the disease [18].

On the other hand, no imaging method can accurately detect pelvic endometriosis with the accuracy needed to replace surgery [2]. Therefore, a thorough understanding of the pathophysiology, implementing non-invasive tools, and discovering new biomarkers are essential for developing novel diagnostic approaches for this debilitating condition [19,20]. 

In recent years, the introduction of omics sciences employing modern technologies has improved the knowledge of several diseases, and it continues to gain importance both in clinical and research settings [21,22]. Genomics and proteomics provided a more complete understanding of some biological events related to gene and protein expression. At the same time, metabolomics is defined as the “quantitative measurement of the dynamic multi-parametric metabolic response of living systems to pathophysiologic stimuli or genetic modification” [23] and offers the possibility to quantify and identify a broad range of endogenous and exogenous low molecular weight metabolites (lipids, amino acids, sugars, biogenic amines, etc.), providing a comprehensive view of metabolic changes during various physiological and pathological processes [24,25]. Small metabolites play a fundamental role in biological systems, representing potential candidates to understand disease phenotypes and pathological mechanisms and impacting the improvement of diagnosis of disease, establishment of therapeutic approach, response to the therapy, and classification of the patients [26]. 

Several analytical techniques can acquire the metabolic profile and simultaneously measure many metabolites or potential biomarkers in a single experiment. Among these, mass spectrometry (MS) and nuclear magnetic resonance (NMR) are the most widely used techniques [27,28,29,30]. NMR is a rapid and nondestructive analytical method that requires simple sample preparation, and spectra can be used to identify several metabolites. However, only the most abundant metabolites are detected because it is relatively insensitive, and millimolar to high micromolar concentrations are often required [31]. Conversely, MS approaches offer combined sensitivity and selectivity platforms for metabolomics analysis [32]. Selectivity among similar metabolites can be ensured by various MS/MS techniques and by combination with separation modules, such as gas chromatography (GC) or liquid chromatography (LC). Several pieces of evidence suggested that a specific metabolomics profile of endometriosis patients resulted from the analysis of the samples with MS techniques, which allowed the measurement of several classes of metabolites, including lipids, in both serum and peritoneal fluid [33,34,35].

The metabolic approach is a valuable and promising way to discover new biomarkers for endometriosis and reveal metabolic pathways associated with disease pathogenesis [20,22,36,37,38,39,40,41]. Because of the unique strength of this field, our study focused on metabolic changes in endometriosis patients (E) using gas chromatography–mass spectrometry (GC–MS) to improve understanding of metabolic pathways involved in endometrial pathogenesis and to provide new functional biomarkers. GC–MS is a high-throughput analytical platform that has been used for untargeted studies of altered metabolism in a variety of applications [28,42]. GC–MS-based metabolomics allow the identification and quantification of small-molecule metabolites (<650 Da), drugs, and toxins, using chemical derivatization to make these compounds volatile for the analysis. Several database annotations using large spectral libraries identify over 200 compounds from human body fluids [43]. As a second point of our study, we aimed to compare the patterns observed in the endometriosis group with those detected in a cohort of asymptomatic women without endometriosis (NE).

## 2. Materials and Methods

### 2.1. Patients and Study Design

This prospective observational study was carried out in the Division of Gynecology and Obstetrics, Department of Surgical Sciences, University of Cagliari, Cagliari, Italy, between November 2015 and June 2016. The trial was registered with the number NCT02337816 and approved by the Duilio Casula University Hospital Ethics Committee, Monserrato, Cagliari (ENDOMETAB01-Prot. 2015/3649).

The manuscript was prepared following the Strengthening the Reporting of Observational Studies in Epidemiology (STROBE) statement [44].

Fifty-four consecutive women, ranging in age from 18 to 50 years (mean age 58.7), who needed surgery for suspected endometriosis or other clinical conditions were enrolled in the study. Exclusion criteria were assessed as follows: hormonal treatment two months before surgery, menopausal status, pregnancy, gynecological cancers, and pelvic inflammatory disease. Before joining the study, each woman provided written informed consent, and the patient’s anonymity was preserved. No study advertising was made, and no remuneration was offered. None of the enrolled patients suffered from autoimmune diseases, nor did they have symptoms referable to potential autoimmune diseases. Clinical data were collected, including socio-demographic data from patients, symptoms, comorbidities, previous therapies, and ultrasound data. 

8–12 h of fasting were observed before surgery, and all women were treated with an osmotic laxative (Isocolan^®^, Giuliani S.p.a., Milano, Italy). Before surgery, each patient’s blood sample was collected in the middle of the follicular phase and sent to the laboratory within twenty minutes. Serum samples were stored at −80 ° C until use. After surgery, we also collected a surgical report to verify the diagnosis. Based on the diagnosis, ten patients were excluded due to the presence of neoplastic or inflammatory pathologies, and the remaining patients were divided into four groups: (1) 22 patients with symptoms and with a histological diagnosis of endometriosis (group E); (2) 10 asymptomatic patients without a diagnosis of endometriosis with uterine fibroids or pelvic organ prolapse (group NE); (3) 4 patients with symptoms but without a diagnosis of endometriosis (4 patients); (4) 3 asymptomatic patients with a diagnosis of endometriosis. The third and fourth groups were not considered for metabolomic analyses due to the low number of patients.

The American Society for Reproductive Medicine classification was used to assess the severity of endometriosis [45].

Considering group E, all patients were classified at stage IV as severe endometriosis and presented the following symptoms before surgery: dysmenorrhea, chronic pelvic pain, dyspareunia, dyschezia, and dysuria. Metabolomic analysis was performed, including groups E and NE, so 37 patients were recruited. Table 1 and Table 2 show all demographic data.

A total of nineteen patients (11 E, 8 NE) showed the presence of comorbidity, nineteen patients (14E and 5 NE) reported previous intake of hormone therapies, thirteen patients (12 E, 1 NE) had ultrasound suggestive of endometriosis, and 22 patients (all belonging to the E class) had a surgical diagnosis of endometriosis.

### 2.2. Sample Preparation

The samples were prepared as described in a previous study [22]. Sera were centrifuged at 4500 rpm for 10 min at 4 °C, and 400 µL of supernatant were transferred to an Eppendorf tube. Hydrophilic and lipophilic metabolites were extracted and separated using a modified Folch method [46]. Four hundred µL of each serum sample was mixed with 600 µL of methanol containing an internal standard of Succinic acid-2,2,3,3-d4 (Sigma-Aldrich, St. Louis, MO, USA), 600 µL of chloroform, and 175 µL of distilled water. Then, they were centrifuged at 4500 rpm for 20 min at 4 °C. The lipid chloroform phase and the water/methanol phase have been separated. 

### 2.3. GC–MS Analysis

A total of 150 µL of hydrophilic phase was dried with speed-vac ON and derivatized as follows: 50 μL of a methoxyamine solution in pyridine (10 mg/mL) (Sigma-Aldrich, St. Louis, MO, USA) was added, and after one hour at 70 °C, 100 μL of N-Methyl-N- (trimethylsilyl)-trifluoroacetamide, MSTFA (Sigma-Aldrich, St. Louis, MO, USA) was also added and left at room temperature for 1 h. The samples were resuspended with 150 μL of hexane (Sigma-Aldrich, St. Louis, MO, USA). A microliter of the derivatized sample was injected split-free into a 7890A gas chromatograph coupled with a 5975C Network mass spectrometer (Agilent Technologies, Santa Clara, CA, USA) equipped with a 30 m × 0.25 mm ID, fused silica capillary column of 30 m 0.25 mm ID, which was chemically bound with 0.25 μM TG-5MS stationary phase 0.25 M TG-5MS (Thermo Fisher Scientific, Waltham, MA, USA). The injector temperature was 250 °C. The gas flow through the column was 1 mL/min. The transfer line temperature was 280 °C. The initial temperature of the column was kept at 50 °C for 3 min, then increased from 50 °C to 250 °C at 10 °C/min and maintained at 250 °C for 12 min. After instrumental analysis, the identification of the metabolites was carried out using the standard NIST 08 (http://www.nist.gov/srd/mslist.cfm (accessed on 16 November 2022)), Fiehn 2013 (http://fiehnlab.ucdavis.edu/Metabolite-Library-2007 (accessed on 16 November 2022)) and GMD (http://gmd.mpimp-golm.mpg.de (accessed on 16 November 2022)) mass spectra libraries (match ≥ 40%) and when available, by comparison with authentic standards. Data processing was performed using a Knime pipeline [47]. In summary, peak detection and deconvolution were performed in an R-XCMS package, filtering using blank samples and keeping features present in ≥50% of the samples. Missing value imputation was performed with the random forest algorithm. Concentrations of discriminant metabolites were obtained by the chromatogram area and then normalized by the total area (=100). 

### 2.4. Multivariate Statistical Analysis

SIMCA-P software (ver. 16.0, Umetrics, Sweden) was used to perform multivariate statistical analysis. First, variables were UV-scaled, and principal component analysis (PCA) was applied. It is essential to explore sample distributions without a priori classification and identify potential outliers through the DmodX and Hotelling T^2^ tests. 

Then, Partial Least Squares Discriminant Analysis (PLS-DA) was applied. It maximizes the discrimination between samples assigned to different classes [48]. Statistical parameters such as variance and predictive ability (R^2^X, R^2^Y, Q^2^) were established to evaluate the suitability of the models. R^2^ and Q^2^ can vary between 0 and 1 values. R^2^ indicates the proportion of variance in the data explained by the model and indicates goodness to fit. Q^2^ indicates the proportion of variance in the data predictable by the model and indicates the goodness of prediction. A good prediction model is achieved when Q^2^ > 0.5 [48].

Furthermore, a permutation test (n = 400) was performed to validate the model. The PLS-DA model scores were tested using CV-ANOVA (ANalysis Of Variance), testing of Cross-Validated predictive residuals, to verify significance (*p* < 0.05). CV-ANOVA is a diagnostic tool for assessing the reliability of the models [49]. The discriminant metabolites were selected by evaluating the loading plot and the VIP value. VIPs of more than 1 are the most relevant terms to explain Y (two classes assignment). 

Univariate statistical analyses of the data were performed using GraphPad Prism software (version 7.01, GraphPad Software, Inc., San Diego, CA, USA). The Mann–Whitney U test was conducted to verify the significance of the metabolic results obtained from multivariate statistical analysis. ROC curves are designed to test the sensitivity and specificity of a selected metabolite pool.

MetaboAnalyst 5.0 (www.metaboanalyst.ca (accessed on 16 November 2022)), a web server designed to obtain comprehensive analysis, visualization, and interpretation of metabolomic data [50], was used to generate metabolic pathways. This method allows a correlation between metabolite changes and metabolic networks.

## 3. Results

This study aimed to investigate serum metabolic changes associated with endometriosis. Therefore, thirty-two serum samples from women were analyzed with GC–MS: twenty-two patients were affected by symptomatic endometriosis, and ten were unaffected. 

Twenty-two metabolites, including organic, amino, fatty, and sugars, were identified in the samples.

**Figure 1:**
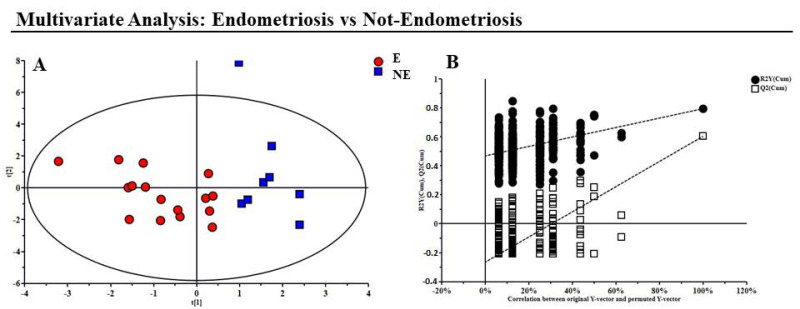
GC−MS analysis of serum Endometriosis (E) and Not−Endometriosis (NE) samples. (**A**) Supervised model PLS−DA score plot from E and NE samples analyzed with the GC−MS; (**B**) Validation of the model via permutation test (*n* = 400).

As a first step, PCA non-supervised analysis was performed, and through the application of the T^2^-Hotelling test, eight outliers (six E and two NE) were identified and excluded from the subsequent analysis. The PLS-DA analysis was then performed to explore the presence of any differences between the NE and pathological E samples (Figure 1A).

The resulting model showed good statistical parameters: R^2^X = 0.45, R^2^Y = 0.731, Q^2^ = 0.453, and *p* = 0.003. Then, the permutation test was used to validate the model: R^2^ intercept = 0.42; Q^2^ intercept = −0.24 (Figure 1B).

The VIP list obtained from the PLS-DA model showed that seven metabolites were the most responsible for separating the two groups, having a VIP value greater than 1. Metabolites were threonic acid, 3-hydroxybutyric acid, proline, alanine, valine, lactate, and phenylalanine. 

**Figure 2:**
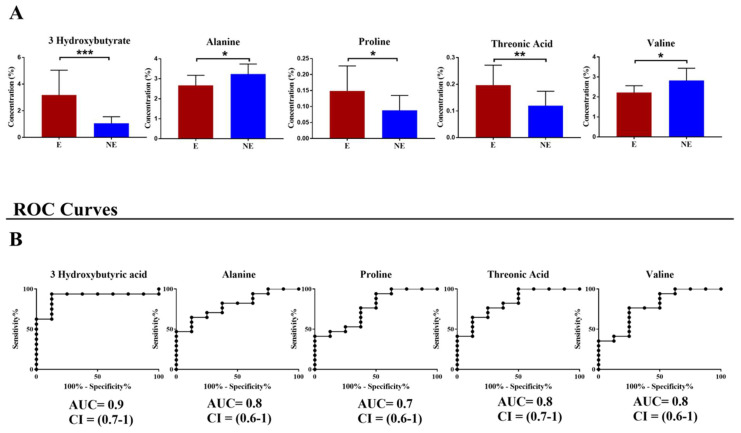
Univariate comparisons between Endometriosis (E) and Not−Endometriosis (NE) samples. (**A**) Graphs of the most important metabolites which changed their concentration with a * *p*−value < 0.05; ** *p*−value <0.01; *** *p*−value < 0.001. The red bar indicates E patients while the blue bar indicates NE patients; (**B**) ROC curves of the discriminant metabolites.

These seven metabolites underwent a Mann–Whitney U test to find significant changes. Only five metabolites showed a *p*-value < 0.05 (Figure 2A) and were used to build the ROC curves to test their sensitivity and specificity as diagnostic biomarkers (Figure 2B). 

Moreover, we performed the combined ROC-curve with the metabolites, which significantly changed their concentration, and we found that the best ROC-curve was obtained combined with 3-OH butyrate, threonic acid, and alanine (AUC = 0.91 CI = 0.8–1, *p*-value = 0.002).

**Figure 3:**
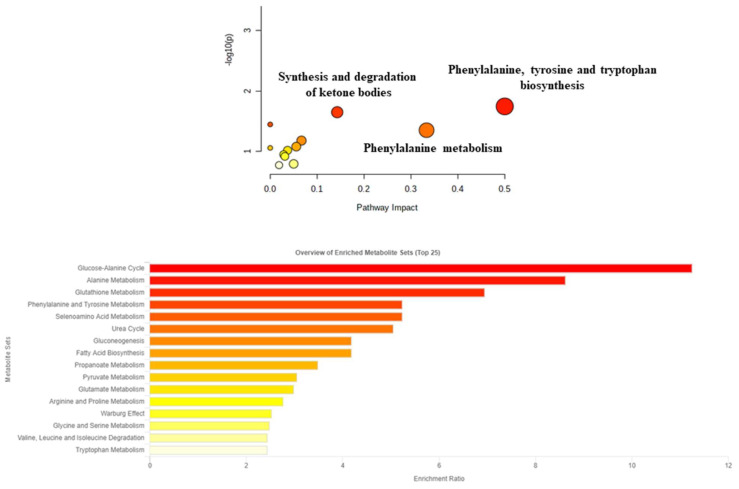
Metabolic pathways built using only metabolites with a VIP value higher than 1.

To better understand which pathways were involved in the disease, all metabolites with a VIP value greater than 1 were used for the pathway analysis (Figure 3). As shown in the figure, the altered pathways were the synthesis and degradation of ketone bodies, the biosynthesis of phenylalanine, tyrosine, tryptophan, and phenylalanine metabolism.

## 4. Discussion

Endometriosis is a well-characterized disease and represents an example of a complex disease in which different factors are involved, such as inflammation, cell invasion and angiogenesis, genetic mutations, steroid hormone metabolism, and oxidative stress [12,14,51,52]. It is well accepted that identifying precise serum biomarkers for endometriosis could avoid the need for laparoscopy, making diagnosing the disease less invasive and more accessible. A non-invasive early diagnosis could have an impact on the management and medical or surgical treatment of the disease [53,54]. The biomarker most consistently studied in endometriosis has been CA125 [55,56,57,58]. Mol and colleagues found that CA125 may be more beneficial in diagnosing advanced stages (III–IV) than stages I and II of the disease [59]. There is increasing evidence that global metabolic profiles are useful for identifying diagnostic biomarkers in easily accessible biofluids [22,60,61,62]. Metabolomics has emerged as an effective tool for monitoring disease progression [63] and distinguishing between diseased and non-diseased states [64].

The present prospective observational translational study is an attempt to identify differently expressed metabolites in serum samples from endometriosis patients using GC–MS-based metabolomics, trying to clarify the pathogenesis of this disease and find new biomarkers for a non-invasive diagnosis. We start from a previous experience in the Division of Gynecology and Obstetrics, University of Cagliari, Italy, to study the identification and quantification of a wide range of metabolites in serum samples from women affected and not affected by endometriosis using ^1^H-NMR [22].

Partially in line with our previous study, the results showed an increase in the concentrations of proline, β-hydroxybutyric acid (a common feature with our past study), and threonic acid, and a decrease in valine and alanine, as well as an alteration of pathways such as biosynthesis and synthesis of phenylalanine, tyrosine, and tryptophan, and degradation of ketone bodies.

The development of fibrosis in endometrial lesions is a complex phenomenon whose fundamental mechanisms are not yet fully understood. Fibrosis is consistently present in all forms of endometriosis, leading to classic symptoms related to the disease, such as pain and infertility [65]. Proline is an important and essential component of collagen and has been shown to fuel protein production, which is needed for cell proliferation [66]. Some previous studies suggest that collagen concentration is significantly higher in chronic endometriosis-associated fibrosis and may play an important role in patients with endometriosis [67]. In addition, Plaka et al. [68] have shown an increase in serum proline levels in these patients. It could be related to increased proline biosynthesis and its increased use in collagen synthesis as support. Consistent with this evidence, our results showed an increase in serum proline levels in patients affected by endometriosis, similar to Dutta et al. [38], who also found a positive correlation between serum proline levels with early (minimal/mild) disease. However, as demonstrated by this latter mentioned study, proline’s trend in endometriosis patients could be controversial, especially considering the different degrees of severity of the disease. Moreover, proline level was decreased in follicular fluids of patients affected by endometriosis compared to control subjects [69].

Oxidative stress, defined as an imbalance between reactive oxygen species (ROS) and antioxidants, can cause general inflammation reactions in the peritoneal cavity and may be involved in the pathophysiology of endometriosis [51,70]. In our study, we found a significant increase in threonic acid in patients with endometriosis: it is a primary product of the oxidative breakdown of ascorbic acid [71]. The elevated serum levels of this sugar acid shown in our study, as well as the altered balance in the concentration of ketone bodies (which are by-products of the glutathione oxidation pathway), further support the fundamental role that oxidative stress plays in the pathogenesis of this disease [72,73]. Furthermore, changes in the ketone body concentration balance may be indirect signs of oxidative stress, as ketone is a by-product of the glutathione oxidation pathway, and the strong relationship between endometriosis and oxidative stress has already been demonstrated [74,75].

Ketone bodies are also involved in energy production. Glucose metabolism fuels 95% or more ATP production, but in case of an energetic challenge, the sources of alternative fuels are ketones. In fact, they provide acetyl coenzyme A (acetyl-CoA), which enters the tricarboxylic acid cycle (TCA) to generate ATP [76]. Bioenergetic metabolism is essential for cells of the immune system to perform its specific functions [77]. A situation of chronic immune activation, such as endometriosis, can exceed the physiological bioenergetic metabolism consuming a considerable amount of energy (up to 2000 kJ/day and more) [78]. 

Furthermore, elevated ketone body β-hydroxybutyric acid levels impaired bone marrow cell proliferation, lymphocyte proliferation in vitro, and in vitro chemotactic differentials of leukocytes in animals [79]. This mechanism could also characterize women with endometriosis. In addition, serum concentrations of β-hydroxybutyric acid were also high in patients with ovarian cancer [80]. Some endometriotic tissue behaviors may be similar to those of cancerous cells, so that some metabolic changes may be shared between the two diseases [81].

We also found a decrease in alanine in the serum of patients affected by endometriosis compared to those not affected. This result is in line with Jana et al.’s study [82], where an increased utilization of this amino acid as a significant gluconeogenic precursor was suggested to meet the high glucose demand and consumption by fast-growing endometrial cells outside of uterine tissue. In the presence of energetic imbalance, alanine blood levels decrease more than any other amino acid, mainly due to the fuel for gluconeogenesis [83]. This phenomenon is driven by the glucose-alanine cycle, where pyruvate is indirectly transformed into alanine through transamination in skeletal muscle. Interestingly, some studies suggested that ketone body infusion lowers L-alanine blood levels more than any other amino acid [84,85]. Despite the fact that we found a decreased level of alanine, other studies found an increased level of it in patients affected by endometriosis compared to control subjects, as well as valine, which we found decreased in endometriosis patients compared to NE [86]. It is a branched-chain amino acid (BCAA) and, together with leucine and isoleucine, can provide carbon precursors useful for synthesizing glucose and other molecules to fuel TCA metabolism and oxidative phosphorylation and provide energy for cells [87]. Furthermore, BCAAs can supply nitrogen nucleotide and protein synthesis [88]. However, the interactions between BCAAs and systemic metabolism are not entirely understood. Still, interesting evidence suggested a role in maintaining immune function and homeostasis [89] with the regulation of several biological processes, such as autophagy and cell growth and proliferation [90]. Furthermore, in vitro studies reported that immune cells use BCAA as substrates, showing an increase in the uptake of these amino acids [91]. As endometriosis is strongly associated with an inflammatory state [14], and the immune system is believed to play a central role in its etiology, pathophysiology [92], and associated morbidities, it is reasonable to expect a decrease in BCAA valine. Our findings are consistent with Santonastaso et al.’s metabolomic study, where a valine level reduction was found in the follicular fluid of endometriosis patients [69], although other studies showed a reverse trend [38,93]. 

Considering the results obtained through the pathway analysis, the biosynthesis of phenylalanine, tyrosine, and tryptophan, together with the metabolism of ketone bodies previously discussed, emerged as the most altered.

The biosynthesis of phenylalanine, tyrosine, and tryptophan is strongly linked to an inflammatory environment [86]. The relationship between the metabolic pathways of phenylalanine and inflammation has been reported in the literature, and it has been observed that during body inflammation, the expression of phenylalanine levels increases. Inflammatory cytokines induce muscle breakdown with the consequent release of phenylalanine as a substrate for gluconeogenesis to supply the increased metabolic demand during inflammation processes [94]. Therefore, a higher phenylalanine concentration could be associated with the catabolic state. 

Tryptophan is involved in two major metabolic pathways: kynurenine and serotonin (5-HT) formation [95]. The kynurenine metabolic pathway (KP) represents about > 90% of peripheral tryptophan metabolism in mammals and plays a crucial role in many biological processes, including immunoregulation [96,97,98]. Dysfunction of immune responses is suggested to promote endometriosis [99] which, together with the presence of inflammation, is correlated with alteration in the kynurenine metabolic pathway with significant changes in the concentrations of its metabolic products [100,101]. The activity of indolamine 2,3-dioxygenase (IDO), the enzyme that promotes tryptophan degradation along the KP, is strongly induced by pro-inflammatory stimuli [102]. It has been proposed that, during inflammation, tryptophan metabolism occurs mainly in immune cells, while in homeostatic/healthy conditions, most TRP degradation occurs in the liver [103]. This can be explained by the increased demand for energy metabolism, redox balance in immune cells, and the requirement of KP metabolites for immune regulatory functions [104,105]. Tryptophan metabolites, 4,6-dihydroxyquinoline, and 5-hydroxy-L-tryptophan were markedly altered in the urine of endometriosis model rats [106]. 

## 5. Conclusions

The results of the present study demonstrated that the application of the GC–MS metabolomics strategy to discover new potential biomarkers has been useful in the endometriosis diagnostic process and in clarifying the disease’s pathophysiology. Indeed, a specific pathological metabolic fingerprint, in line with our previous work, was established. Furthermore, increased concentrations of proline, β-hydroxybutyric acid, and threonic acid and a decrease in valine and alanine were found in the endometriosis patients compared to the non-endometriosis group. This paper describes a translational study where the common clinical practice “marries” the basic research with innovative analytical and statistical techniques. 

However, despite the innovative nature of this study, it shows some limitations. First, the number of enrolled patients is relatively small, and further studies are needed with a larger cohort of patients, possibly with the inclusion of a group of healthy control subjects to better define the pathological metabolic profile of patients affected by endometriosis. Second, the analysis of the mean age of the two classes of patients showed a *p*-value < 0.05. Despite the fact that we didn’t find a correlation between age and the metabolomics profile, this could be considered a confounding factor. Furthermore, it could be desirable for the combination of different analytical platforms to amplify the panel of the metabolites, which could be candidates as biomarkers to improve the clinical management of the patients affected by endometriosis. In this light, NMR spectroscopy, LC–MS, and GC–MS can give complementary information.

Finally, considering that fibrosis represents a hallmark in the endometriosis scenario, the lack of the evaluation of its level in the patients and the correlation with the metabolic changes represent a weak point of this study, as well as the lack of the measure of the enzymatic activity relative to the pathways that we found altered, which could confirm their involvement in the pathological processes. 

In conclusion, literature data about the metabolic changes in patients affected by endometriosis are still conflicting. For this reason, further studies are needed to better clarify this interesting topic.

## Figures and Tables

**Table 1:** Clinical data of the patients affected by severe endometriosis.

Clinical Variables	Group Endometriosis (*n* = 22)
Dysmenorrhea (VAS median [range])	8 (7–10)
Chronic pelvic pain (VAS median, [range])	7 (5–9)
Dyspareunia (VAS median, [range])	8 (7–10)
Dischezia (VAS median, [range])	3 (0–4)
Dysuria (VAS median, [range])	1 (0–5)
Surgical variables	
Single ovarian endometrioma (n; %)	18; 81.8%
Measurements in cm (median, [range])	7 (5–9)
Bilateral ovarian endometrioma (n; %)	6; 27.3%
Measurements in cm (median, [range])	4 (4–6)
Deep infiltrating endometriosis (DIE) (n; %)	22; 100%

VAS: Visual Analogue Scale; DIE: deep infiltrating endometriosis.

**Table 2:** Demographic data of the enrolled patients.

Variables	Endometriosis (*n* = 22)	No Endometriosis (*n* = 10)	*p*-Value
Age (median, range)	34 (22–43)	41 (33–50)	<0.05
BMI (median, range)	20.4 (18.5–29)	23.4 (19.5–31.2)	NS
Ethnicity			
Caucasian	22	10	
Age at menarche, mean	11.7 (1.4)	11.8 (1.5)	NS
Marital status %			
Married	64	50	NS
Divorced	18	20	NS
Single	18	30	NS
Parity%	18	20	NS
One	13.5	20	NS
two	4.5	-	NS
Cigarette smoking%			
Non smoker	77.3	80	NS
Current smoker daily	22.7	20	
Cigarettes (mean ± SD)	10.6 ± 6.2	9.9 ± 6.1	NS
Schooling %			
Elementary and middle school	13.6	20	NS
High school	60	60	NS
Higher education	26.4	20	NS

BMI: Body Mass Index; NS = not significant.

## Data Availability

The data presented in this study are available on request from the corresponding author. The data are not publicly available due to the protection of patient privacy.

## References

[1] Angioni S. (2017). New insights on endometriosis. Minerva Obstet. Gynecol..

[2] Melis G.B., Neri M., Corda V., Malune M.E., Piras B., Pirarba S., Guerriero S., Orrù M., D’Alterio M.N., Angioni S. (2016). Overview of elagolix for the treatment of endometriosis. Expert Opin. Drug Metab. Toxicol..

[3] Angioni S., Cofelice V., Pontis A., Tinelli R., Socolov R. (2014). New trends of progestins treatment of endometriosis. Gynecol. Endocrinol..

[4] Zajec V., Mikuš M., Vitale S.G., D’Alterio M.N., Gregov M., Šarić M.J., Carugno J., Angioni S., Ćorić M. (2022). Current status and challenges of drug development for hormonal treatment of endometriosis: A systematic review of randomized control trials. Gynecol. Endocrinol..

[5] Eskenazi B., Warner M.L. (1997). Epidemiology of endometriosis. Obstet. Gynecol. Clin. N. Am..

[6] Melis I., Agus M., Pluchino N., Sardo A.D.S., Litta P., Melis G.B., Angioni S. (2014). Alexithymia in Women with Deep Endometriosis? A Pilot Study. J. Endometr. Pelvic Pain Disord..

[7] Melis I., Penna M.P., Murru M., Pontis A., Agus M., Angioni S. In Multidimensional assessment of pain in women with endo-metriosis: Preliminary results of the experience in Cagliari. Proceedings of the 2016 IEEE International Symposium on Medical Measurements and Applications (MeMeA).

[8] Stochino-Loi E., Pontis A., Cofelice V., Pirarba S., Fais M.F., Daniilidis A., Melis I., Paoletti A.M., Angioni S. (2019). Effect of ultramicronized-palmitoylethanolamide and co-micronized palmitoylethanolamide/polydatin on chronic pelvic pain and quality of life in endometriosis patients: An open-label pilot study. Int. J. Women’s Health.

[9] D’Alterio M.N., Saponara S., Agus M., Laganà A.S., Noventa M., Loi E.S., Feki A., Angioni S. (2021). Medical and surgical interventions to improve the quality of life for endometriosis patients: A systematic review. Gynecol. Surg..

[10] Angioni S., Cela V., Sedda F., Loi E.S., Cofelice V., Pontis A., Melis G.B. (2015). Focusing on surgery results in infertile patients with deep endometriosis. Gynecol. Endocrinol..

[11] Laganà A.S., Vitale S.G., Salmeri F.M., Triolo O., Frangež H.B., Vrtačnik-Bokal E., Stojanovska L., Apostolopoulos V., Granese R., Sofo V. (2017). Unus pro omnibus, omnes pro uno: A novel, evidence-based, unifying theory for the pathogenesis of endometriosis. Med. Hypotheses.

[12] Angioni S., D’Alterio M.N., Coiana A., Anni F., Gessa S., Deiana D. (2020). Genetic Characterization of Endometriosis Patients: Review of the Literature and a Prospective Cohort Study on a Mediterranean Population. Int. J. Mol. Sci..

[13] Deiana D., Gessa S., Anardu M., Daniilidis A., Nappi L., D’Alterio M.N., Pontis A., Angioni S. (2019). Genetics of endometriosis: A comprehensive review. Gynecol. Endocrinol..

[14] Jiang L., Yan Y., Liu Z., Wang Y. (2016). Inflammation and endometriosis. Front. Biosci..

[15] Velho R.V., Taube E., Sehouli J., Mechsner S. (2021). Neurogenic Inflammation in the Context of Endometriosis-What Do We Know?. Int. J. Mol. Sci..

[16] Viganó D., Zara F., Pinto S., Loddo E., Casula L., Soru M.B., D’Ancona G., D’Alterio M.N., Giuliani C., Angioni S. (2020). How is small bowel permeability in endometriosis patients? a case control pilot study. Gynecol. Endocrinol..

[17] Dorien F.O., Flores I., Waelkens E., D’Hooghe T. (2018). Noninvasive diagnosis of endometriosis: Review of current peripheral blood and endometrial biomarkers. Best Pract. Res. Clin. Obstet. Gynaecol..

[18] Fuldeore M.J., Soliman A.M. (2016). Prevalence and Symptomatic Burden of Diagnosed Endometriosis in the United States: National Estimates from a Cross-Sectional Survey of 59,411 Women. Gynecol. Obstet. Investig..

[19] D’Alterio M.N., Giuliani C., Scicchitano F., Lagana A.S., Oltolina N.M., Sorrentino F., Nappi L., Orru G., Angioni S. (2021). Possible role of microbiome in the pathogenesis of endometriosis. Minerva Obstet. Gynecol..

[20] Angioni S., Saponara S., Succu A.G., Sigilli M., Scicchitano F., D’Alterio M.N. (2021). Metabolomic Characteristics in Endome-triosis Patients. Endometriosis Pathogenesis, Clinical Impact and Management.

[21] Fulghesu A.M., Piras C., Dessì A., Succu C., Atzori L., Pintus R., Gentile C., Angioni S., Fanos V. (2021). Urinary Metabolites Reveal Hyperinsulinemia and Insulin Resistance in Polycystic Ovarian Syndrome (PCOS). Metabolites.

[22] Murgia F., Angioni S., D’Alterio M.N., Pirarba S., Noto A., Santoru M.L., Tronci L., Fanos V., Atzori L., Congiu F. (2020). Metabolic Profile of Patients with Severe Endometriosis: A Prospective Experimental Study. Reprod. Sci..

[23] Nicholson J.K., Lindon J.C. (2008). Systems biology: Metabonomics. Nature.

[24] Boja E.S., Kinsinger C.R., Rodriguez H., Srinivas P. (2014). Integration of omics sciences to advance biology and medicine. Clin. Proteom..

[25] Syggelou A., Iacovidou N., Atzori L., Xanthos T., Fanos V. (2012). Metabolomics in the Developing Human Being. Pediatr. Clin. N. Am..

[26] Arakaki A.K., Skolnick J., McDonald J.F. (2008). Marker metabolites can be therapeutic targets as well. Nature.

[27] Murgia F., Muroni A., Puligheddu M., Polizzi L., Barberini L., Orofino G., Solla P., Poddighe S., Del Carratore F., Griffin J.L. (2017). Metabolomics As a Tool for the Characterization of Drug-Resistant Epilepsy. Front. Neurol..

[28] Papadimitropoulos M.-E.P., Vasilopoulou C.G., Maga-Nteve C., Klapa M.I. (2018). Untargeted GC-MS Metabolomics. Methods Mol. Biol..

[29] Tokarz J., Adamski J., Rižner T.L. (2020). Metabolomics for Diagnosis and Prognosis of Uterine Diseases? A Systematic Review. J. Pers. Med..

[30] Wang R., Li B., Lam S.M., Shui G. (2020). Integration of lipidomics and metabolomics for in-depth understanding of cellular mechanism and disease progression. J. Genet. Genom..

[31] Emwas A.H. (2015). The strengths and weaknesses of NMR spectroscopy and mass spectrometry with particular focus on metabolomics research. Methods Mol. Biol..

[32] Kim S.J., Song H.E., Lee H.Y., Yoo H.J. (2021). Mass Spectrometry-based Metabolomics in Translational Research. Adv. Exp. Med. Biol..

[33] Letsiou S., Peterse D.P., Fassbender A., Hendriks M.M., Broek N.J.V.D., Berger R., O D.F., Vanhie A., Vodolazkaia A., Van Langendonckt A. (2017). Endometriosis is associated with aberrant metabolite profiles in plasma. Fertil. Steril..

[34] Loy S.L., Zhou J., Cui L., Tan T.Y., Ee T.X., Chern B.S.M., Chan J.K.Y., Lee Y.H. (2021). Discovery and validation of peritoneal endometriosis biomarkers in peritoneal fluid and serum. Reprod. Biomed. Online.

[35] Vouk K., Ribič-Pucelj M., Adamski J., Rižner T.L. (2016). Altered levels of acylcarnitines, phosphatidylcholines, and sphingomyelins in peritoneal fluid from ovarian endometriosis patients. J. Steroid Biochem. Mol. Biol..

[36] May K.E., Conduit-Hulbert S.A., Villar J., Kirtley S., Kennedy S.H., Becker C.M. (2010). Peripheral biomarkers of endometriosis: A systematic review. Hum. Reprod. Updat..

[37] Ghazi N., Arjmand M., Akbari Z., Mellati A.O., Saheb-Kashaf H., Zamani Z. (2016). (1)H NMR- based metabolomics approaches as non- invasive tools for diagnosis of endometriosis. Int. J. Reprod. BioMed..

[38] Dutta M., Singh B., Joshi M., Das D., Subramani E., Maan M., Jana S.K., Sharma U., Das S., Dasgupta S. (2018). Metabolomics reveals perturbations in endometrium and serum of minimal and mild en-dometriosis. Sci. Rep..

[39] Lee Y.H., Cui L., Fang J., Chern B.S.M., Tan H.H., Chan J.K.Y. (2016). Limited value of pro-inflammatory oxylipins and cytokines as circulating biomarkers in endometriosis—a targeted ‘omics study. Sci. Rep..

[40] Vicente-Munoz S., Morcillo I., Puchades-Carrasco L., Paya V., Pellicer A., Pineda-Lucena A. (2015). Nuclear magnetic resonance metabolomic profiling of urine provides a noninvasive alternative to the identification of biomarkers associated with endo-metriosis. Fertil. Steril..

[41] Cordeiro F.B., Cataldi T.R., Perkel K.J., do Vale Teixeira da Costa L., Rochetti R.C., Stevanato J., Eberlin M.N., Zylbersztejn D.S., Cedenho A.P., Lo Turco E.G. (2015). Lipidomics analysis of follicular fluid by ESI-MS reveals potential biomarkers for ovarian endometriosis. J. Assist. Reprod. Genet..

[42] Honour J.W. (2006). Gas chromatography-mass spectrometry. Methods Mol. Biol..

[43] Fiehn O. (2016). Metabolomics by Gas Chromatography–Mass Spectrometry: Combined Targeted and Untargeted Profiling. Curr. Protoc. Mol. Biol..

[44] von Elm E., Altman D.G., Egger M., Pocock S.J., Gotzsche P.C., Vandenbroucke J.P., Initiative S. (2014). The Strengthening the Reporting of Observational Studies in Epidemiology (STROBE) Statement: Guidelines for reporting observational studies. Int. J. Surg..

[45] Haas D., Shebl O., Shamiyeh A., Oppelt P. (2012). The rASRM score and the Enzian classification for endometriosis: Their strengths and weaknesses. Acta Obstet. et Gynecol. Scand..

[46] Folch J., Lees M., Sloane Stanley G.H. (1957). A simple method for the isolation and purification of total lipides from animal tissues. J. Biol. Chem..

[47] Liggi S., Hinz C., Hall Z., Santoru M.L., Poddighe S., Fjeldsted J., Atzori L., Griffin J.L. (2018). KniMet: A pipeline for the pro-cessing of chromatography-mass spectrometry metabolomics data. Metabolomics.

[48] Trygg J., Holmes .A.E., Lundstedt .T. (2007). Chemometrics in Metabonomics. J. Proteome Res..

[49] Eriksson L., Trygg J., Wold S. (2008). CV-ANOVA for significance testing of PLS and OPLS® models. J. Chemom..

[50] Xia J., Sinelnikov I.V., Han B., Wishart D.S. (2015). MetaboAnalyst 3.0—Making metabolomics more meaningful. Nucleic Acids Res..

[51] Vitale S.G., Capriglione S., Peterlunger I., La Rosa V.L., Vitagliano A., Noventa M., Valenti G., Sapia F., Angioli R., Lopez S. (2018). The Role of Oxidative Stress and Membrane Transport Systems during Endometriosis: A Fresh Look at a Busy Corner. Oxidative Med. Cell. Longev..

[52] Angioni S., Nappi L., Sorrentino F., Peiretti M., Daniilidis A., Pontis A., Tinelli R., D’Alterio M.N. (2021). Laparoscopic treatment of deep endometriosis with a diode laser: Our experience. Arch. Gynecol. Obstet..

[53] Angioni S., Pontis A., Cela V., Sedda F., Genazzani A.D., Nappi L. (2015). Surgical technique of endometrioma excision impacts on the ovarian reserve. Single-port access laparoscopy versus multiport access laparoscopy: A case control study. Gynecol. Endocrinol..

[54] Stochino-Loi E., Darwish B., Mircea O., Touleimat S., Millochau J.C., Abo C., Angioni S., Roman H. (2017). Does preoperative antimullerian hormone level influence postoperative pregnancy rate in women undergoing surgery for severe endometriosis?. Fertil. Steril..

[55] Socolov R., Butureanu S., Angioni S., Sindilar A., Boiculese L., Cozma L., Socolov D. (2010). The value of serological markers in the diagnosis and prognosis of endometriosis: A prospective case–control study. Eur. J. Obstet. Gynecol. Reprod. Biol..

[56] Mohamed M.L., El Behery M.M., Mansour S.A. (2013). Comparative study between VEGF-A and CA-125 in diagnosis and fol-low-up of advanced endometriosis after conservative laparoscopic surgery. Arch. Gynecol. Obstet..

[57] Matalliotakis I.M., Goumenou A.G., Mulayim N., Karkavitsas N., Koumantakis E.E. (2004). High concentrations of the CA-125, CA 19-9 and CA 15-3 in the peritoneal fluid between patients with and without endometriosis. Arch. Gynecol. Obstet..

[58] Vodolazkaia A., El-Aalamat Y., Popovic D., Mihalyi A., Bossuyt X., Kyama C.M., Fassbender A., Bokor A., Schols D., Huskens D. (2012). Evaluation of a panel of 28 biomarkers for the non-invasive diagnosis of endometriosis. Hum. Reprod..

[59] Mol B.W., Bayram N., Lijmer J.G., Wiegerinck M.A., Bongers M.Y., van der Veen F., Bossuyt P.M. (1998). The performance of CA-125 measurement in the detection of endometriosis: A meta-analysis. Fertil. Steril..

[60] Patel N.R., McPhail M.J., Shariff M.I., Keun H.C., Taylor-Robinson S.D. (2012). Biofluid metabonomics using (1)H NMR spec-troscopy: The road to biomarker discovery in gastroenterology and hepatology. Expert Rev. Gastroenterol. Hepatol..

[61] Weiss R.H., Kim K. (2011). Metabolomics in the study of kidney diseases. Nat. Rev. Nephrol..

[62] Rhee E.P., E Gerszten R. (2012). Metabolomics and Cardiovascular Biomarker Discovery. Clin. Chem..

[63] Muthubharathi B.C., Gowripriya T., Balamurugan K. (2021). Metabolomics: Small molecules that matter more. Mol. Om..

[64] Stoop M.P., Coulier L., Rosenling T., Shi S., Smolinska A.M., Buydens L., Ampt K., Stingl C., Dane A., Muilwijk B. (2010). Quantitative proteomics and metabolomics analysis of normal human cerebrospinal fluid samples. Mol. Cell. Proteom..

[65] Vigano P., Candiani M., Monno A., Giacomini E., Vercellini P., Somigliana E. (2017). Time to redefine endometriosis including its pro-fibrotic nature. Hum. Reprod..

[66] Tanner J.J., Fendt S.-M., Becker D.F. (2018). The Proline Cycle As a Potential Cancer Therapy Target. Biochemistry.

[67] Jussila T., Kauppila S., Bode M., Tapanainen J., Risteli J., Risteli L., Kauppila A., Stenbäck F. (2004). Synthesis and maturation of type I and type III collagens in endometrial adenocarcinoma. Eur. J. Obstet. Gynecol. Reprod. Biol..

[68] Palka J., Oscilowska I., Szoka L. (2021). Collagen metabolism as a regulator of proline dehydrogenase/proline oxidase-dependent apoptosis/autophagy. Am. Acids.

[69] Santonastaso M., Pucciarelli A., Costantini S., Caprio F., Sorice A., Capone F., Natella A., Iardino P., Colacurci N., Chiosi E. (2017). Correction: Metabolomic profiling and biochemical evaluation of the follicular fluid of endometriosis patients. Mol. Biosyst..

[70] Lee Y.H., Yang J.X., Allen J.C., Tan C.S., Chern B.S.M., Tan T.Y., Tan H.H., Mattar C.N.Z., Chan J.K.Y. (2018). Elevated peritoneal fluid ceramides in human endometriosis-associated infertility and their effects on mouse oocyte maturation. Fertil. Steril..

[71] Thomas M., Hughes R. (1983). A relationship between ascorbic acid and threonic acid in guinea-pigs. Food Chem. Toxicol..

[72] Ansariniya H., Yavari A., Javaheri A., Zare F. (2022). Oxidative stress-related effects on various aspects of endometriosis. Am. J. Reprod. Immunol..

[73] Maignien C., Santulli P., Kateb F., Caradeuc C., Marcellin L., Pocate-Cheriet K., Bourdon M., Chouzenoux S., Batteux F., Bertho G. (2020). Endometriosis phenotypes are associated with specific serum metabolic profiles determined by pro-ton-nuclear magnetic resonance. Reprod. Biomed. Online.

[74] Kanikarla-Marie P., Jain S.K. (2015). Hyperketonemia (acetoacetate) upregulates NADPH oxidase 4 and elevates oxidative stress, ICAM-1, and monocyte adhesivity in endothelial cells. Cell. Physiol. Biochem..

[75] Kolb H., Kempf K., Röhling M., Lenzen-Schulte M., Schloot N.C., Martin S. (2021). Ketone bodies: From enemy to friend and guardian angel. BMC Med..

[76] Cunnane S.C., Trushina E., Morland C., Prigione A., Casadesus G., Andrews Z.B., Beal M.F., Bergersen L.H., Brinton R.D., de la Monte S. (2020). Brain energy rescue: An emerging therapeutic concept for neurodegenerative disorders of ageing. Nat. Rev. Drug Discov..

[77] Buttgereit F., Burmester G.-R., Brand M.D. (2000). Bioenergetics of immune functions: Fundamental and therapeutic aspects. Immunol. Today.

[78] Straub R.H., Cutolo M., Buttgereit F., Pongratz G. (2010). Energy regulation and neuroendocrine-immune control in chronic in-flammatory diseases. J. Intern. Med..

[79] Kaufmann T.B., Drillich M., Tenhagen B.A., Heuwieser W. (2010). Correlations between periparturient serum concentrations of non-esterified fatty acids, beta-hydroxybutyric acid, bilirubin, and urea and the occurrence of clinical and subclinical post-partum bovine endometritis. BMC Vet. Res..

[80] Hilvo M., de Santiago I., Gopalacharyulu P., Schmitt W.D., Budczies J., Kuhberg M., Dietel M., Aittokallio T., Markowetz F., Denkert C. (2016). Accumulated Metabolites of Hydroxybutyric Acid Serve as Diagnostic and Prognostic Biomarkers of Ovarian High-Grade Serous Carcinomas. Cancer Res.

[81] Ishikawa M., Nakayama K., Nakamura K., Ono R., Sanuki K., Yamashita H., Ishibashi T., Minamoto T., Iida K., Razia S. (2018). Affinity-purified DNA-based mutation profiles of endometriosis-related ovarian neoplasms in Japanese patients. Oncotarget.

[82] Jana S.K., Dutta M., Joshi M., Srivastava S., Chakravarty B., Chaudhury K. (2013). 1H NMR Based Targeted Metabolite Profiling for Understanding the Complex Relationship Connecting Oxidative Stress with Endometriosis. BioMed. Res. Int..

[83] Felig P., Owen O.E., Wahren J., Cahill G.F. (1969). Amino acid metabolism during prolonged starvation. J. Clin. Investig..

[84] Sherwin R.S., Hendler R.G., Felig P. (1976). Effect of diabetes mellitus and insulin on the turnover and metabolic response to ketones in man. Diabetes.

[85] Soto-Mota A., Norwitz N.G., Evans R.D., Clarke K. (2022). Exogenous d-beta-hydroxybutyrate lowers blood glucose in part by decreasing the availability of L-alanine for gluconeogenesis. Endocrinol. Diabetes Metab..

[86] Dutta M., Joshi M., Srivastava S., Lodh I., Chakravarty B., Chaudhury K. (2012). A metabonomics approach as a means for identification of potential biomarkers for early diagnosis of endometriosis. Mol. Biosyst..

[87] Sivanand S., Heiden M.G.V. (2020). Emerging Roles for Branched-Chain Amino Acid Metabolism in Cancer. Cancer Cell.

[88] Ananieva E.A., Wilkinson A.C. (2018). Branched-chain amino acid metabolism in cancer. Curr. Opin. Clin. Nutr. Metab. Care.

[89] Ananieva E.A., Powell J.D., Hutson S.M. (2016). Leucine Metabolism in T Cell Activation: mTOR Signaling and Beyond. Adv. Nutr. Int. Rev. J..

[90] Bonvini A., Coqueiro A.Y., Tirapegui J., Calder P.C., Rogero M.M. (2018). Immunomodulatory role of branched-chain amino acids. Nutr. Rev..

[91] Li P., Yin Y.-L., Li D., Kim S.W., Wu G. (2007). Amino acids and immune function. Br. J. Nutr..

[92] Vallvé-Juanico J., Houshdaran S., Giudice L.C. (2019). The endometrial immune environment of women with endometriosis. Hum. Reprod. Updat..

[93] Strasser B., Sperner-Unterweger B., Fuchs D., Gostner J.M. (2016). Mechanisms of Inflammation-Associated Depression: Immune Influences on Tryptophan and Phenylalanine Metabolisms. Curr. Top. Behav. Neurosci..

[94] Luporini R.L., Pott-Junior H., Leal M.C.B.D.M., Castro A., Ferreira A.G., Cominetti M.R., Anibal F.D.F. (2021). Phenylalanine and COVID-19: Tracking disease severity markers. Int. Immunopharmacol..

[95] Zhou X.-H., Zhang A.-H., Wang L., Tan Y.-L., Guan Y., Han Y., Sun H., Wang X.-J. (2016). Novel chinmedomics strategy for discovering effective constituents from ShenQiWan acting on ShenYangXu syndrome. Chin. J. Nat. Med..

[96] Trabanelli S., Ocadlikova D., Evangelisti C., Parisi S., Curti A. (2011). Induction of Regulatory T Cells by Dendritic Cells through Indoleamine 2,3- dioxygenase: A Potent Mechanism of Acquired Peripheral Tolerance. Curr. Med. Chem..

[97] Song H., Park H., Kim Y.-S., Kim K.D., Lee H.-K., Cho D.-H., Yang J.-W., Hur D.Y. (2011). l-Kynurenine-induced apoptosis in human NK cells is mediated by reactive oxygen species. Int. Immunopharmacol..

[98] Zaher S.S., Germain C., Fu H., Larkin D.F., George A.J. (2011). 3-hydroxykynurenine suppresses CD4+ T-cell proliferation, in-duces T-regulatory-cell development, and prolongs corneal allograft survival. Investig. Ophthalmol. Vis. Sci..

[99] Osuga Y., Koga K., Hirota Y., Hirata T., Yoshino O., Taketani Y. (2010). Lymphocytes in Endometriosis. Am. J. Reprod. Immunol..

[100] Wang Q., Liu D., Song P., Zou M.H. (2015). Tryptophan-kynurenine pathway is dysregulated in inflammation, and immune ac-tivation. Front. Biosci..

[101] Urata Y., Koga K., Hirota Y., Akiyama I., Izumi G., Takamura M., Nagai M., Harada M., Hirata T., Yoshino O. (2014). IL-1β Increases Expression of Tryptophan 2,3-dioxygenase and Stimulates Tryptophan Catabolism in Endometrioma Stromal Cells. Am. J. Reprod. Immunol..

[102] Gunther J., Fallarino F., Fuchs D., Wirthgen E. (2020). Editorial: Immunomodulatory Roles of Tryptophan Metabolites in Inflam-mation and Cancer. Front. Immunol..

[103] Moffett J.R., Arun P., Puthillathu N., Vengilote R., Ives J.A., Badawy A.A.-B., Namboodiri A.M. (2020). Quinolinate as a Marker for Kynurenine Metabolite Formation and the Unresolved Question of NAD+ Synthesis During Inflammation and Infection. Front. Immunol..

[104] Biefer H.R.C., Vasudevan A., Elkhal A. (2017). Aspects of Tryptophan and Nicotinamide Adenine Dinucleotide in Immunity: A New Twist in an Old Tale. Int. J. Tryptophan Res..

[105] Grant R.S. (2018). Indoleamine 2,3-Dioxygenase Activity Increases NAD+ Production in IFN-gamma-Stimulated Human Primary Mononuclear Cells. Int. J. Tryptophan Res..

[106] Wu X.-H., Zhao C., Zhang A.-H., Zhang J.-Q., Wang X., Sun X.-L., Sun Z., Wang X.-J. (2018). High-throughput metabolomics used to identify potential therapeutic targets of Guizhi Fuling Wan against endometriosis of cold coagulation and blood stasis. RSC Adv..

